# Early life shifts in cortical inhibitory–excitatory balance underlies sensitive periods and skill development

**DOI:** 10.3389/fncel.2026.1808258

**Published:** 2026-06-03

**Authors:** Dekel Taliaz

**Affiliations:** Taliaz, Petah Tikva, Israel

**Keywords:** cortical plasticity, early brain development, excitation inhibition balance, GABAergic signaling, inhibitory interneurons, neurogenesis, sensitive periods, skill learning

## Abstract

Early human development is characterized by sensitive periods which impact long-term cognitive and behavioral outcomes. While these windows of heightened plasticity are well documented, the cellular mechanisms that enable and regulate them remain incompletely understood. In this conceptual article, I propose that early-life shifts in cortical inhibitory–excitatory balance, driven by prolonged neurogenesis, migration, and maturation of GABAergic interneurons, play a central role in opening, shaping, and closing sensitive periods and thereby guide skill development. Drawing on evidence from human and animal studies, I synthesize findings showing that inhibitory interneurons are integrated into cortical circuits well into postnatal life, where they regulate intrinsic and sensory-driven activity, sculpt synaptic connectivity, coordinate interactions with glial cells, and progressively refine network dynamics. The developmental strengthening of inhibition alters excitation–inhibition ratios, drives the transition from highly synchronous early activity to decorrelated and efficient adult-like firing patterns, and gates critical period plasticity across cortical regions. I argue that these inhibitory processes are not merely stabilizing but actively facilitate learning by suppressing non-relevant activity and enabling the emergence of specialized functional networks. This framework highlights fundamental differences between infant and adult learning mechanisms and suggests that individual variability in inhibitory circuit development may underlie differences in cognitive trajectories and vulnerability to neurodevelopmental disorders. Together, this synthesis positions early inhibitory interneuron development as a key mechanistic substrate linking sensitive periods to lifelong skill acquisition and behavioral individuality.

## 1 Introduction

From birth, infants are naturally tuned to multisensory events, integrating information across senses to form coherent representations of the ever-changing environment; a capacity that enables adaptation and survival (Tierney and Nelson, 2009; Zhao et al., 2019; Nava et al., 2024). As neurodevelopment unfolds over the first years of life, it is characterized by multiple sensitive periods: time windows during which specific experiences exert a distinctive influence on immediate and long-term neurobehavioral outcomes (Bruer, 2001; Knudsen, 2004; Zhao et al., 2019; Luby et al., 2020; Sullivan and Opendak, 2020). Critical periods are a specific subtype of sensitive periods - narrower window in which specific experience is required for normal development; and if this input is absent, neural function is permanently altered and later experience cannot fully compensate (Knudsen, 2004). Together, these periods are crucial for the development of the sensory, social, and cognitive systems, abstract concepts and body representation, and are supported by early neural specialization (Elston and Timms, 1992; Cashdan, 1994; Paterson et al., 2006; Uylings, 2006; Zeanah et al., 2011; Dehaene-Lambertz and Spelke, 2015; Feldman, 2015; Gao et al., 2015; Harris and Mason, 2017; Sullivan and Opendak, 2020; Thompson and Steinbeis, 2020; Gee and Cohodes, 2021; Röder et al., 2021).

Disruption of inhibitory circuits might be responsible for some of the clinical features of disorders such as schizophrenia, autism and intellectual disabilities and it is gaining support, as summarized in (Marín, 2012; Contractor et al., 2021). Studies in humans and in animal models indicate that more-subtle perturbations in the excitatory–inhibitory (E/I) balance exist in multiple psychiatric conditions (Selten et al., 2018). For example, it was shown that altered FoxG1 expression in both excitatory and inhibitory neurons disrupts neural circuit development and leads to autism-like social deficits in mouse models. These findings identify the second postnatal week as a critical developmental window during which precise FoxG1 regulation is necessary for normal social behavior (Miyoshi et al., 2021). Disruption of the development or function of GABAergic interneurons in the cerebral cortex leads to epilepsy and may contribute to the emergence of specific symptoms of certain neuropsychiatric disorders, in particular those associated with cognitive impairment (Marín, 2012). In addition, in mouse models of temporal lobe epilepsy, aberrant maturation of adult-born granule cells during an early critical period contributes to hippocampal circuit reorganization and seizure development. Temporarily silencing these cells during this window reduces structural abnormalities, normalizes circuit connectivity, and decreases spontaneous recurrent seizures, highlighting a GABA-dependent activity mechanism in epileptogenesis (Lybrand et al., 2021). Abnormal development of fast-spiking parvalbumin-expressing (PV+) interneurons may predispose individuals to schizophrenia. In autism spectrum disorders, disruption of the E/I balance may occur in several neural systems, including the neocortex, the basal ganglia and the hindbrain. Increasing evidence indicates that abnormal GABAergic function is linked to several other neurological conditions, including Angelman’s syndrome, fragile X syndrome and neurofibromatosis type I. The fact that cortical interneurons are genetically specified early during development might be particularly relevant to neuropsychiatric disorders, because many of these conditions emerge early in life and are thought to be caused by defects in brain development (Marín, 2012).

Importantly, later learning builds on the neural infrastructure established during these sensitive periods, with foundational wiring that support more efficient and flexible adaptation to novel experiences throughout life (Zhao et al., 2019; Luby et al., 2020; van Vugt et al., 2021). For example, while first language is acquired during sensitive period where structural and functional changes establish specialized, long-lasting neuronal networks, second language learners rarely achieve native-like proficiency, especially in phonology and syntax, due to reduced plasticity and reliance on alternative neural pathways (White et al., 2013; Pant et al., 2020; Siahaan, 2022). Although adults learning second language show neuroplastic changes, including increased gray and white matter in language-related brain regions, these changes are less pronounced and less efficient than those seen with early language exposure (Li et al., 2014; Liu et al., 2020; Wei et al., 2024). Similarly, while core language areas such as Broca’s and Wernicke’s regions are engaged for both the first and second languages, second language learning requires greater activation in cognitive control networks to help manage interference and competition between languages (Perani and Abutalebi, 2005; Abutalebi, 2008; Calabria et al., 2018; Li and Jeong, 2020).

In a former paper (Taliaz, 2013), I have suggested that neurogenesis in the infant human brain plays an essential role in the rapid development and sensitive periods occurring during the first years of life; a suggestion that was partially supported by observations that new neurons continue to be generated postnatally in the subgranular zone of the dentate gyrus of the human hippocampus (Sorrells et al., 2018). In the same context, although not reflecting neurogenesis per se, one study reported extensive migration and integration of young neurons, which move tangentially along the walls of the lateral ventricles and blood vessels before dispersing over long distances into cortical regions, where they differentiate and contribute to inhibitory circuits in the infant human frontal lobe (Paredes et al., 2016). In my former paper (Taliaz, 2013), I focused on the association between structural changes and skills development, arguing that the addition of new neurons would bring with it an addition of synapses, dendritic arbors and axons, all of which may contribute to the large increases in gray and white matter that are unique to these periods. In the present paper, I propose that widespread neurogenesis, migration of progenitor neurons into the cortex, and differentiation into inhibitory interneurons, plays a crucial role in the mechanisms underlying sensitive periods and skill development. I would argue that these processes expand the pool of cortical inhibitory neurons, which fine-tune developing networks during the sensitive periods and thereby shape the individual’s lifelong behavioral patterns. These inhibitory neurons are not only regulators of circuit refinement but also active facilitators of learning, by selectively suppressing non-relevant activity, and thereby guiding the emergence of specialized functional patterns. This view is supported by the abundance and diversity of inhibitory neurons in humans (Wei et al., 2025), which is greater compared to species such as rodents and reflect evolutionary pressures for complex network modulation (Petanjek et al., 2009; Marín, 2025). In addition, this view highlights the profoundly different mechanisms underlying learning in infants and adults. As reviewed below, during infancy the brain exhibits heightened plasticity, characterized by robust neurogenesis, synaptic formation, and the differentiation of inhibitory interneurons. In contrast, adult learning primarily involves molecular-level modifications at existing neurons, such as changes in synaptic strength and receptor composition, with key mechanism of synaptic plasticity at excitatory synapses (Kennedy, 2016; Gazerani, 2025).

In order to establish this opinion, the article adopts a targeted, theory-driven approach to literature selection, prioritizing foundational studies, high-impact empirical findings, and recent reviews that directly inform the proposed mechanistic framework. Rather than providing a systematic or exhaustive review, the aim is to integrate converging evidence across developmental, cellular, and systems neuroscience to support a conceptual account of early learning. Importantly, several of the topics discussed remain under active debate, most notably the extent and functional significance of human postnatal neurogenesis. Where relevant, such controversies are explicitly acknowledged, and the interpretation presented here reflects one plausible synthesis of the current evidence rather than a consensus view. Emphasis is placed on well-established mechanisms, while more speculative elements are presented with appropriate caution. This approach is intended to balance conceptual clarity with transparency regarding evidential limits.

### 1.1 Early neurodevelopment

Neurodevelopment during the first years of the human life is governed by interacting and overlapping frameworks of cellular growth, synaptic remodeling, genetic and epigenetic regulation, and environmental modulation (Cioni et al., 2016; Krebs et al., 2017; Kundakovic and Jaric, 2017; van Dyck and Morrow, 2017; Guyer et al., 2018; Natu et al., 2021; Reh and Zimmer-Bensch, 2021; Chini et al., 2022; Menassa et al., 2022; Hanson et al., 2024; Sorrells, 2024). These include the generation of brain cells through neurogenesis, migration of cells to their proper locations, differentiation into unique cell types, establishment of connections with other neurons by extending dendrites and axons and by forming synapses, refining and eliminating excess connections during maturation and pruning, and enhancing communication efficiency through supportive structures and myelination (Committee on Integrating the Science of Early Childhood Development, 2000). These neuronal and supportive mechanisms drives large-scale network organization, during which the foundations of sensory processing, motor coordination, and higher-order cognition are established (Huttenlocher and Dabholkar, 1997; Committee on Integrating the Science of Early Childhood Development, 2000; Taliaz, 2013; Dennis et al., 2016; Kostović et al., 2019). At the same time, synaptic contacts that are not included in neuronal circuits are gradually eliminated (Huttenlocher and Dabholkar, 1997). The developing brain generates more neurons than needed and programmed cell death (apoptosis) eliminates excess neurons, optimizing network connectivity and function (Pfisterer and Khodosevich, 2017; Fricker et al., 2018; Wong and Marín, 2019). Survival mechanisms vary by neuron type and brain region, with specific transcription factors and signaling pathways influencing vulnerability and timing of cell death (Pfisterer and Khodosevich, 2017; Wong and Marín, 2019). One such mechanism is the competition for limited survival-promoting molecules (neurotrophins) released by target tissues, glia, or neighboring neurons, with only those that successfully obtain enough trophic support survive (Clarke, 1985; Davies, 2003; Nikoletopoulou et al., 2010; Dekkers et al., 2013).

These mechanisms collectively enable the rapid and adaptive maturation of brain circuits and neural networks foundational for later cognitive and behavioral functions. For example, the distinction between experience-expectant and experience-dependent plasticity emphasizes both the universal developmental processes that rely on typical environmental input and the unique adaptations that reflect individual experience (Greenough et al., 1987). Complementarily, Hebbian principles of synaptic strengthening (“cells that fire together wire together”) highlight the importance of temporally correlated activity in shaping neural circuits and large-scale network organization (Hebb, 1949). In addition, silent or poorly connected neurons are more prone to die, while neurons with higher spontaneous electrical activity and successful integration into networks are more likely to survive (Tashiro et al., 2006; Wong Fong Sang et al., 2021; Warm et al., 2022). This so called spontaneous activity - the electrical activity of a neuron in the absence of any external sensory or known input - is not random, often follows structured patterns of activity, and can involve large-scale coordinated cascades across neuronal populations (van Pelt et al., 2004; Mazzoni et al., 2007; Guo et al., 2020; Liu et al., 2021; Swindale et al., 2023). The underlying mechanisms involve intrinsic ionic currents, such as persistent sodium and cationic currents, as well as the interplay of excitatory and inhibitory synaptic pathways (Raman et al., 2000; Häusser et al., 2004; Jackson et al., 2004). Synaptic mechanisms involving N-methyl-D-aspartate (NMDA) and *γ*-aminobutyric acid (GABA) receptors modulate the pattern and synchrony of spontaneous firing, particularly in network settings, and astrocytes can further regulate firing modes and burst dynamics (Mazzoni et al., 2007; Teplov et al., 2021; Palabas et al., 2022). Hence, in following mentioning I will address this activity as intrinsic rather than spontaneous.

According to the view presented here, these developmental processes, ranging from activity-dependent synaptic refinement and network formation to selective neuronal survival, critically depend on the timely neurogenesis and maturation of inhibitory interneurons, which enable the stabilization and functional organization of emerging neural circuits.

### 1.2 Inhibitory neurons in early neurodevelopment

It is estimated that there are over 20 different subtypes of GABAergic interneurons in the cortex, but studies performed in rodents suggested that three subtypes account for most of the population. These include two types of PV-expressing interneurons, a heterogeneous population of somatostatin (SST)-expressing interneurons, and heterogeneous population of the ionotropic serotonin receptor 5HT3a (5HT3aR)-expressing interneurons (Kelsom and Lu, 2013); which divide into Vasoactive Intestinal Peptide (VIP)-expressing and non-VIP expressing subtypes (Georgiou et al., 2022). The various cortical interneuron subtypes have distinct or only partially overlapping morphological, electrophysiological and neurochemical characteristics (Marín, 2012). SST interneurons develop earlier than PV + cells and play a role in establishing early circuit balance and dendritic inhibition (Mòdol et al., 2024), the maturation of PV + interneurons is linked to critical period plasticity (Matsumoto et al., 2025), while the function of the 5HT3aR group is modulatory and include inhibition of other inhibitory neurons (Pi et al., 2013).

#### 1.2.1 Neurogenesis, migration and differentiation

Cortical neurons can be broadly categorized into glutamate-releasing excitatory neurons, and GABA-releasing inhibitory neurons (Delgado et al., 2022). Extensive migration of young neurons in the human frontal lobe continues for several months after birth, with the majority of these cells expressing markers of inhibitory interneurons (Paredes et al., 2016). This integration of inhibitory interneurons is thought to support the maturation and plasticity of the frontal cortex, a region critical for social behavior and executive function. Indeed, disruption of this migration is linked to neurodevelopmental disorders, suggesting its importance for normal cognitive development and possibly for early learning capacity (Paredes et al., 2016). These inhibitory neurons integrate into cortical circuits, where the numbers and subtypes are shaped by both genetic programs and activity-dependent mechanisms (Petanjek et al., 2009; Paredes et al., 2016; Warm et al., 2022; Marín, 2025). Together with the proposed cortical neurogenesis, these processes determines the total pool of neurons, including both excitatory and inhibitory types, while individual cortical progenitors can generate both types and thus provide the basis for neuronal diversity and network complexity (Petanjek et al., 2009; Delgado et al., 2022). Extended neurogenesis of neural progenitor cells, which differentiate into inhibitory neurons, may allow prolonged addition of interneurons, increasing network complexity and plasticity (Kim and Paredes, 2021). GABAergic signaling in the cerebral cortex plays an important role in regulating early developmental processes such as migration and differentiation (Luhmann et al., 2014) and possibly neurogenesis (Jurkowski et al., 2020). Ionotropic GABA_A_ receptors mediate fast inhibitory synaptic transmission through intrinsic chloride channels. Their activation enhances chloride conductance, leading to hyperpolarization and a reduction in neuronal excitability. In contrast, metabotropic GABA_B_ receptors, located at both pre- and postsynaptic sites, are G-protein-coupled and regulate slow inhibitory neurotransmission via second messenger pathways that modulate calcium and potassium channel activity (Zhang et al., 2007).

The survival of inhibitory interneurons in the developing cortex is highly dependent on the inputs they receive from excitatory (pyramidal) neurons. Interneurons that receive strong excitatory input during a critical postnatal window are more likely to survive, while those with lower activity are more prone to apoptosis (Wong et al., 2018). On the same time, as shown in animal models of cerebral ischemia and seizures, GABAergic activation can promote neuroprotection by counterbalance excessive glutamatergic activity and by that to reduce excitotoxic neuronal death [e.g., (Zhang et al., 2007; Wei et al., 2012)]. GABAergic interneurons also play a critical role in shaping and synchronizing early cortical network activity, initially exerting excitatory effects before transitioning to inhibition as networks mature (Luhmann et al., 2014, 2016; Kirmse and Zhang, 2022; Warm et al., 2022). For example, it was shown in a mouse model that GABAergic neurons in the CA1 hippocampus are excitatory at postnatal day 3, driving most of the intrinsic firing of pyramidal cells at that stage, but shift to an inhibitory role by postnatal day 7 (Murata and Colonnese, 2020). The underlying mechanism for the transition of GABAergic interneurons from excitatory to inhibitory involves changes in the intracellular chloride ion concentration within developing neurons. Early in development, neurons express high levels of the NKCC1 chloride importer, leading to elevated intracellular chloride. When GABA_A_ receptors are activated under these conditions, chloride flows out of the cell, causing depolarization and thus excitatory effects. As the brain matures, the expression of the KCC2 chloride exporter increases, lowering intracellular chloride levels; now, GABA_A_ receptor activation results in chloride influx, hyperpolarizing the neuron and producing inhibitory effects (Maffei et al., 2017; Warm et al., 2022; Lozovaya et al., 2023; McArdle et al., 2024).

Collectively, these findings indicate that early cortical development and network maturation may critically relies on the neurogenesis, migration, survival, and functional integration of inhibitory interneurons, whose regulation of excitability and synchronization is essential for shaping plasticity, protecting developing circuits, and supporting the emergence of higher-order cognitive functions.

#### 1.2.2 Neuronal firing

Inhibitory cells play essential role in the establishment of early firing patterns, which initially include both intrinsic and synchronous firing, and that later undergo refinement and become more specialized and functionally efficient through mechanisms of activity-dependent plasticity (Luhmann et al., 2016). These early firing patterns are essential for refining thalamocortical circuits and suppressing programmed cell death, supporting the formation of cortical columns and microcircuits (Luhmann and Khazipov, 2018; Mizuno et al., 2018; Molnár et al., 2020). The development of inhibitory circuits parallels the appearance of correlated oscillatory patterns, such as delta brushes (Warm et al., 2022), which are neuronal bursts that are critical for brain maturation, serving as a foundation for later, more complex activity patterns (Arichi et al., 2017; Luhmann and Khazipov, 2018; Schaworonkow and Voytek, 2021). Weaker inhibition or increased electrical coupling (gap junctions) can promote synchrony, allowing neurons to fire in coordinated patterns or oscillations (Hansel and Mato, 2003; Devalle et al., 2017; di Volo and Torcini, 2018; Todd et al., 2025). Increasing inhibitory synaptic strength or the E/I ratio promotes asynchronous firing by tightly balancing excitatory inputs, reducing correlations between neurons, and leading to irregular, independent spiking (Zhang et al., 2011; Devalle et al., 2017; di Volo and Torcini, 2018; Li and Shew, 2020; Chini et al., 2022). This E/I ratio, established by the coordinated development of inhibitory and excitatory synapses, is vital for healthy network activity (Fossati et al., 2016; Parodi et al., 2023; Horton et al., 2024). Inhibitory plasticity and interneuron diversity further modulate these transitions, enabling flexible control of network dynamics (Wu et al., 2022; Bocchio et al., 2024).

Interneuron subtypes are generated and integrated over a prolonged period, extending late into gestation and postnatally. The migration and proposed extended neurogenesis allows gradual refinement of E/I ratio and region-specific modulation of firing, especially in higher associative cortices (Kim and Paredes, 2021; Lindhout et al., 2024). Hence, early-born inhibitory neurons critically control when and how developing neurons fire, shaping the transition from synchronous, bursting activity to sparse, decorrelated adult-like firing patterns.

#### 1.2.3 Synaptogenesis and network refining

Inhibitory neurons are fundamental to synaptogenesis and network refinement in early human infancy, guiding circuit maturation synapse formation and dynamic functional shifts. Interneuron neurogenesis and migration are unusually protracted into early postnatal life, likely allowing extended refinement of cortical neural networks and sensitivity to environment (Kim and Paredes, 2021; Marín, 2025). In the same manner, synaptogenesis begins around the 20th gestational week and continue throughout childhood and adolescence (Tau and Peterson, 2010; Uytun, 2018). The timing and pattern of GABAergic synaptogenesis are crucial for establishing proper neural network architecture, while disruptions in this process can lead to long-term behavioral abnormalities (Molnár et al., 2020; Magno et al., 2021; Abusaada et al., 2025). In most developing neurons, GABA induces localized calcium elevations in neurites and growth cones, while activity-dependent GABA release provides developing neurons with essential cues for growth and maturation (Ji et al., 2024). Early intrinsic and sensory-driven activity, modulated by GABAergic neurons, is essential for the maturation of functional circuits and the pruning of excess connections (Luhmann et al., 2016; Molnár et al., 2020; Warm et al., 2022). Inhibitory synapses initially act as scaffolds to stabilize growing excitatory connections, helping to balance network activity and prevent hyperexcitability (Fossati et al., 2016). Nevertheless, timely clearance of excess cortical interneurons is critical for correct functional maturation of circuits that drive adult behavior (Magno et al., 2021). Thereby, synaptogenesis and network refinement in early development critically depend on the presumed neurogenesis and timely integration of inhibitory interneurons, which mature into stable and functional neural circuits.

#### 1.2.4 Supportive structure formation and interaction

Postnatal modulation of glial cells by inhibitory neurons is a dynamic, reciprocal process essential for healthy brain development. Glial cells play complementary and stage-dependent roles in orchestrating the development and maintenance of inhibitory networks during critical periods, through coordinated regulation of synapse formation, stabilization, and elimination (Dzyubenko and Hermann, 2023). Astrocytes regulate synaptogenesis, stabilize synaptic connectivity, and dynamically modulate chloride homeostasis, thereby influencing inhibitory neurotransmission and overall inhibitory tone (Dzyubenko and Hermann, 2023; Untiet et al., 2023). Microglia are described as contributing to activity-dependent synaptic pruning, selectively eliminating synapses and thereby shaping circuit maturation and E/I balance (Andoh et al., 2019; Patel et al., 2019; Dzyubenko and Hermann, 2023). In addition, astrocytes support neuronal homeostasis and excitability through gliotransmitter release and neuroinflammatory signaling, further modulating E/I dynamics (Sood et al., 2021; Onat et al., 2025). Neuronal GABAergic signaling shapes microglial function, glia influences inhibitory circuit maturation, and both cell types coordinate to refine synaptic networks and maintain neural balance. For example, early-born inhibitory interneurons release GABA that directly drives astrocyte morphogenesis (Cheng et al., 2023). Oligodendrocytes and inhibitory neurons engage in reciprocal signaling that guides myelination, such that the pattern and extent of myelination on inhibitory axons are determined by both intrinsic neuronal properties and extrinsic cues from oligodendrocytes (Zonouzi et al., 2019; Benamer et al., 2020; Serrano-Regal et al., 2020; Fletcher et al., 2021). In addition, microglia express GABA receptors and selectively interact with inhibitory synapses during critical postnatal periods (Favuzzi et al., 2021). GABA signaling triggers transcriptional programs in microglia, enabling them to sculpt inhibitory connectivity without affecting excitatory synapses. Here again, disruption of this pathway impairs circuit development and leads to behavioral abnormalities (Nakayama et al., 2018; Favuzzi et al., 2021). Thus, the formation and maturation of supportive neural structures may depend on the neurogenesis and functional integration of inhibitory interneurons, whose GABAergic signaling orchestrates reciprocal interactions with glial cells to regulate myelination, synaptic refinement, and the maintenance of balanced and resilient neural circuits.

#### 1.2.5 Sensitive periods

Neurogenesis, differentiation and maturation into GABA-containing cells and networks has been identified as a key regulator of critical and sensitive periods plasticity, acting as a developmental switch that both enables and constrains windows of heightened neuroplasticity (Hensch, 2005). Inhibitory neurons are fundamental in establishing, timing, and closing sensitive periods, with a threshold level of inhibition appears to be essential to open the critical period, and modulating inhibitory circuit maturation can shift the timing of critical periods (Fletcher et al., 2021). These inhibitory processes play a central role in the frontal cortex, where E/I ratio contributes to the emergence of executive functions, attentional control, and the early foundations of goal-directed behavior (Diamond, 2002). Modulation of the local E/I ratio via pharmacological intervention, sensory experience, stress, nutritional status, or genetic and epigenetic factors can alter the temporal dynamics of critical period windows in both animal models and humans (Reh et al., 2020). For example, antidepressant treatments have been shown to modulate cortical excitability by altering glutamatergic and GABAergic neurotransmission, thereby influencing excitation-inhibition balance in mood-related circuits (Musazzi et al., 2013; Duman et al., 2019); and prenatal exposure to antidepressants and depressed maternal mood alter trajectory of infant speech perception (Weikum et al., 2012). In addition, environmental enrichment rescues binocular matching of orientation preference in mice that have a precocious critical period due to genetically enhanced inhibition (Wang et al., 2013). Tightly regulated E/I ratio in the adult cortex arises from pronounced shifts in the relative strength of inhibitory, but not excitatory synapses during the critical period, with local inhibitory structural changes serving as the foundation for altered E/I balance across postnatal development (Zhang et al., 2011). The same inhibitory circuits that track critical periods timing generate moment-by-moment oscillatory activity in the brain, which are potential biomarker of plasticity level in the circuit, allowing more precise determination of critical periods trajectories and individual differences (Reh et al., 2020). For example, neuronal circuits in the primary visual cortex are shaped by experience during ‘critical periods’, which are triggered by the functional maturation of local inhibitory connections and driven by a specific, late-developing subset of PV + interneurons (Hensch, 2005). Abnormalities in early GABAergic development or network integration are linked to neurodevelopmental and psychiatric disorders (Petanjek et al., 2009; Magno et al., 2021), while the fine-tuning of neuronal networks by GABAergic neurons during sensitive periods underlies the foundation for individual behavioral traits and cognitive functions (Melzer and Monyer, 2020; Molnár et al., 2020; Magno et al., 2021).

**Figure 1:**
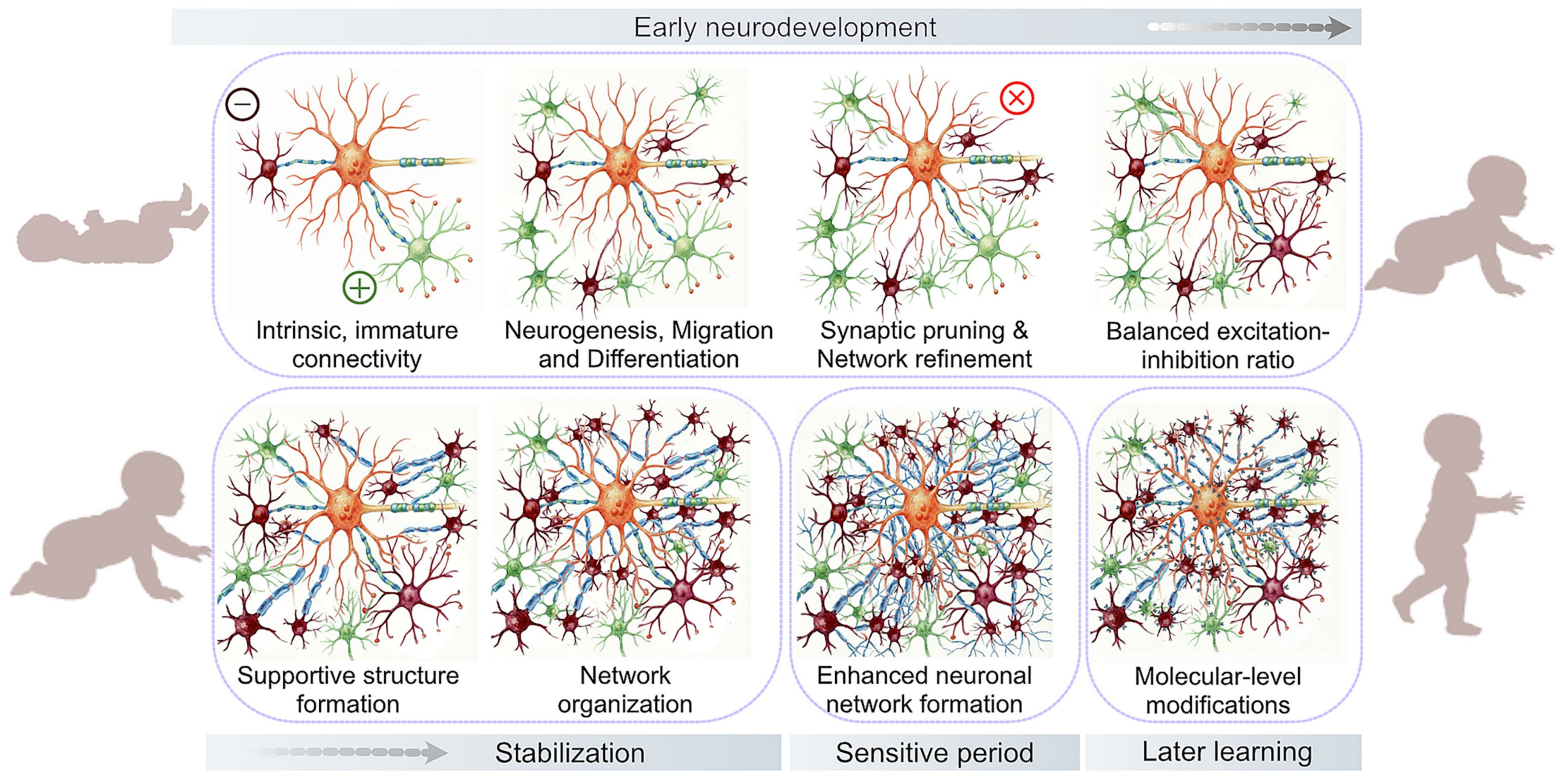
Conceptual illustration of neurodevelopment across early life. Developmental progression from early infancy to toddlerhood is accompanied by increasing complexity and integration of neuronal networks. Early stages are characterized by intrinsic and immature connectivity, followed by rapid neurogenesis, neuronal migration, and differentiation of excitatory (green cells) and inhibitory (dark red cells) neurons, processes that are strongly promoted by early GABAergic signaling. Inhibitory interneurons also play a central role in both network expansion and in subsequent refinement through synaptic pruning, the formation of supportive structure formation (myelination, indicated in blue), and establishment of excitation–inhibition (E/I) balance. The relative strength of inhibition increases with age, resulting in a reduced E/I ratio, which underlies the transition from early highly synchronous activity patterns to later decorrelated neural dynamics. The maturation of inhibitory networks has been identified as a key regulator of critical period plasticity, acting as a developmental switch that both enables and constrains windows of heightened neuroplasticity. These processes lay the foundation for later learning, cognitive flexibility, and adaptive behavior by shaping the efficiency and large-scale organization of neural circuits. Note that this schematic represents a simplification; many of these processes co-occur and may manifest at different times across brain regions, developmental stages, and functions. The orange cell indicates neuron that receive both inhibitory and excitatory inputs (e.g., pyramidal or motor cells).

Overall, the opening, progression, and closure of sensitive periods critically depend on the proposed neurogenesis, as well as differentiation and maturation of inhibitory interneurons, whose developmentally regulated control of E/I ratio and oscillatory dynamics gates plasticity, shapes experience-dependent circuit refinement, and lays the foundation for long-term cognitive and behavioral outcomes unique to each individual (Figure 1).

Developmental progression from early infancy to toddlerhood is accompanied by increasing complexity and integration of neuronal networks. Early stages are characterized by intrinsic and immature connectivity, followed by rapid neurogenesis, neuronal migration, and differentiation of excitatory (green cells) and inhibitory (dark red cells) neurons, processes that are strongly promoted by early GABAergic signaling. Inhibitory interneurons also play a central role in both network expansion and in subsequent refinement through synaptic pruning, the formation of supportive structure formation (myelination, indicated in blue), and establishment of excitation–inhibition (E/I) balance. The relative strength of inhibition increases with age, resulting in a reduced E/I ratio, which underlies the transition from early highly synchronous activity patterns to later decorrelated neural dynamics. The maturation of inhibitory networks has been identified as a key regulator of critical period plasticity, acting as a developmental switch that both enables and constrains windows of heightened neuroplasticity. These processes lay the foundation for later learning, cognitive flexibility, and adaptive behavior by shaping the efficiency and large-scale organization of neural circuits. Note that this schematic represents a simplification; many of these processes co-occur and may manifest at different times across brain regions, developmental stages, and functions. The orange cell indicates neuron that receive both inhibitory and excitatory inputs (e.g., pyramidal or motor cells).

#### 1.2.6 Energy considerations

Interneurons inhibit neuronal activity as early as the embryonic phase, but the relative strength of inhibition increases with age and the addition of inhibitory cells through neurogenesis in early life, resulting in a reduced E/I ratio. This developmental shift underlies the transition from early highly synchronous activity patterns to later decorrelated neural dynamics - an activity regime that supports efficient information storage and retrieval while minimizing energy expenditure (Chini et al., 2022). Inhibitory interneurons, especially fast-spiking types like PV + cells, play a crucial role in balancing excitation and inhibition in neural circuits. However, despite their high firing rates, these interneurons achieve remarkable energy efficiency due to the complementary tuning of sodium and potassium channel gating, which minimizes ion flux overlap during action potentials, keeping energy use close to the theoretical minimum (Hu et al., 2018; Gotti et al., 2021). High mitochondrial content and oxidative enzyme expression further support their metabolic needs (Kann et al., 2014; Kann, 2016; Gotti et al., 2021). Inhibitory interneurons synchronize principal neuron activity, enabling efficient information processing through gamma oscillations (30–100 Hz). This rhythmic inhibition is essential for sensory perception, memory, and motor behavior, and it allows for the formation of cooperative neuronal assemblies, optimizing the transmission and routing of excitatory activity (Kann et al., 2014; Roux and Buzsáki, 2015; Kann, 2016). Gamma oscillation is a rhythmic brain activity that emerges early in development but undergo significant maturation before stabilizing in adulthood. Early gamma oscillations are considered immature forms of adult gamma rhythms and are crucial for the development of cortical circuits underlying cognitive functions; disruptions in this maturation are linked to neuropsychiatric disorders (Bitzenhofer, 2023). In mice, fast oscillatory activity appears prominently during the second postnatal week, initially around 15 Hz, and accelerates to the adult gamma frequency range by the fourth postnatal week, paralleling maturation of inhibitory interneurons that regulate E/I balance (Bitzenhofer et al., 2020; Bitzenhofer, 2023). Human studies show a developmental trajectory where gamma power and phase-locking increase from childhood into adolescence, peaking around mid-adolescence, then decline slightly toward early adulthood, reflecting processes like synaptic pruning and increasing GABAergic inhibition (Cho et al., 2015; McKeon et al., 2023). Additionally, developmental changes include shifts from low-amplitude broadband gamma in children to higher-amplitude band-limited gamma in adults, associated with a decreasing E/I signaling ratio in cortical microcircuits (Rhodes et al., 2025).

Efficient interneuron function prevents excessive energy consumption by maintaining network stability and preventing runaway excitation (Tsodyks et al., 1997; Buzsáki et al., 2007). This function resonance efficient coding theories, which suggest that neuronal networks are optimized to maximize informational value while minimizing metabolic costs, including in infants (Laughlin, 2001; Triesch, 2013). Taken together, the developmental optimization of neural energy efficiency depends on the proposed neurogenesis, as well as maturation of inhibitory interneurons, whose progressive strengthening and metabolically efficient firing regulates E/I ratio, stabilize network dynamics, and support information-rich yet energetically economical brain activity.

## 2 Future perspectives

The framework advanced here positions early-life shifts in cortical E/I balance as a central mechanism linking neurodevelopmental dynamics to sensitive periods and long-term skill acquisition. Moving forward, several conceptual and empirical directions are likely to be particularly informative for refining this model and extending its implications.

First, future work will benefit from greater temporal resolution in tracking inhibitory circuit development in humans. Most current knowledge relies on cross-sectional postmortem data or indirect imaging proxies (Ament et al., 2023; Marín, 2025). Longitudinal approaches that integrate advanced neuroimaging, electrophysiological markers, and molecular profiling may allow more precise mapping of how inhibitory neuron integration, subtype composition, and functional maturation unfold across infancy and early childhood (Ahmad et al., 2022; Fiske et al., 2025). For example, future work could combine methods such as magnetic resonance spectroscopy (MRS)–derived GABA measurements (Yanez Lopez et al., 2021), EEG indices of inhibitory function (Ünsal et al., 2024), peripheral transcriptomic profiling to capture developmental changes in interneuron-related gene expression (Singer et al., 2026), resting-state fMRI connectivity (Mongerson et al., 2017), magnetoencephalography (MEG) measures of oscillatory dynamics (Rhodes et al., 2025) and epigenetic profiling (Hodge et al., 2024). Such approaches could clarify whether distinct sensitive periods are governed by shared inhibitory mechanisms or by region- and circuit-specific trajectories of inhibitory maturation.

Second, an important challenge lies in linking cellular-level inhibitory processes to large-scale network dynamics and behavior (Shine et al., 2021). While growing evidence connects inhibitory maturation to changes in oscillatory activity and E/I balance, future studies should aim to explicitly relate these neural signatures to emerging cognitive and behavioral capacities. Identifying developmental neural markers that index the opening, progression, and closure of sensitive periods could provide a mechanistic bridge between cellular neurodevelopment and observable learning trajectories. Prior work has already begun to link neural signatures of inhibitory circuit function to cognitive development. For example, longitudinal EEG studies have shown that resting frontal gamma power in infancy and early childhood is strongly associated with concurrent language ability, attention, and inhibitory control, suggesting that oscillatory markers of inhibition track emerging cognitive functions (Benasich et al., 2008). Similarly, task-based infant EEG studies demonstrate that gamma-band responses during social perception paradigm reflect domain-specific cognitive processing, providing early evidence that inhibitory-related oscillations are functionally tied to cognition (Grossmann et al., 2007). More recently, longitudinal designs combining early electrophysiology with later behavioral assessments have shown that infant neural measures can predict later cognitive outcomes, including executive function and inhibitory control (Zhang et al., 2024). Building on these approaches, future studies could more directly integrate multimodal neural measures with repeated, domain-specific cognitive testing to map how the maturation of inhibitory circuits constrains the emergence of distinct cognitive abilities over development.

Third, individual variability represents a critical but underexplored dimension. Genetic, epigenetic, metabolic, and environmental factors are all likely to influence the pace and pattern of inhibitory neuron development (Kann, 2016; Reh et al., 2020; Reh and Zimmer-Bensch, 2021; Marín, 2025). Future research that integrates these sources of variability may help explain why sensitive periods differ in timing and impact across individuals, and why some developmental trajectories confer resilience whereas others increase vulnerability to neurodevelopmental and psychiatric disorders.

Finally, this perspective invites a reframing of early learning and plasticity. Rather than viewing sensitive periods as passive windows of heightened excitability, future models may conceptualize them as actively regulated states, shaped by the gradual strengthening and specialization of inhibitory circuits. This view emphasizes inhibition not as a constraint on plasticity, but as a dynamic organizer that enables efficient learning, stabilizes emerging representations, and optimizes energy use in developing networks.

Together, these directions suggest that advancing our understanding of inhibitory neuron development will be essential for a more complete theory of human neurodevelopment. By integrating cellular, circuit, and system-level approaches, future work can further elucidate how early inhibitory processes sculpt the brain’s capacity for learning, adaptation, and behavioral individuality across the lifespan.

## 3 Conclusion

The first years of human life represent a period of intense neurodevelopment, during which rapidly expanding circuits are refined into increasingly specialized and efficient networks. Infant learning relies on the migration and differentiation of neurons, with neurogenesis proposed to be an essential factor underlying the sensitive periods in early life. Here I introduced the next step of the mechanism of early-life learning, in which neurogenesis, migration and differentiation into GABAergic interneurons play a central role in shaping early neural network activity and the conditions for efficient learning. This inhibitory regulation is not merely supportive but formative: it governs the timing of sensitive periods, shapes the trajectory of cortical specialization, and provides the foundation for higher-order cognitive functions. In the frontal cortex for example, inhibitory processes enable the emergence of executive control, attentional regulation, and the earliest forms of goal-directed behavior. Together, these findings underscore the critical importance of inhibitory neurons generated in the first years of life, in early brain maturation. Far from acting only as circuit stabilizers, they serve as dynamic facilitators of learning and adaptation during sensitive periods, guiding the development of neural networks. These capacities underly lifelong behavioral and cognitive capacities and form the base for the distinctive behavioral profiles of individuals.

## Data Availability

The original contributions presented in the study are included in the article/supplementary material, further inquiries can be directed to the corresponding author/s.
